# Elective parotidectomy and neck dissection are not beneficial in cutaneous squamous cell carcinoma of the head^☆^

**DOI:** 10.1016/j.bjorl.2023.101352

**Published:** 2023-10-23

**Authors:** Zuzana Horakova, Ivo Starek, Richard Salzman

**Affiliations:** University Hospital Olomouc and Faculty of Medicine and Dentistry of Palacky University Olomouc, Department of Otorhinolaryngology and Head and Neck Surgery, Olomouc, Czechia

**Keywords:** Skin cancer, Cutaneous squamous cell carcinoma, Elective parotidectomy, Elective neck dissection, Occult metastasis

## Abstract

**Objective:**

Cutaneous Squamous Cell Carcinoma (cSCC), a tumor with a significantly increasing incidence, is mostly diagnosed in the head region, where tumors have a worse prognosis and a higher risk of metastases. The presence of metastases reduces specific five-year survival from 99% to 50%. As the risk of occult metastases does not exceed 10%, elective dissection of the tributary parotid and neck lymph nodes is not recommended.

**Methods:**

We retrospectively analyzed a group of 12 patients with cSCC of the head after elective dissections of regional (parotid and cervical) nodes by means of superficial parotidectomy and selective neck dissection.

**Results:**

We diagnosed occult metastases neither in the cervical nor parotid nodes in any patient. None were diagnosed as a regional recurrence during the follow-up period.

**Conclucion:**

Our negative opinion on elective parotidectomy and neck dissection in cSCC of the head is in agreement with the majority of published studies. These elective procedures are not indicated even for tumors showing the presence of known (clinical and histological) risk factors for lymphogenic spread, as their positive predictive value is too low. Elective parotidectomy is individually considered as safe deep surgical margin. If elective parotidectomy is planned it should include only the superficial lobe. Completion parotidectomy and elective neck dissection are done in rare cases of histologically confirmed parotid metastasis in the parotid specimen. Preoperatively diagnosed parotid metastases without neck involvement are sent for total parotidectomy and elective selective neck dissection. Cases of clinically evident neck metastasis with no parotid involvement, are referred for comprehensive neck dissection and elective superficial parotidectomy. The treatment of concurrent parotid and cervical metastases includes total conservative parotidectomy and comprehensive neck dissection.

**Level of evidence:**

How common is the problem?

Step 4 (Case-series)

Is this diagnostic or monitoring test accurate? (Diagnosis)

Step 4 (poor or non-independent reference standard)

What will happen if we do not add a therapy? (Prognosis)

Step 4 (Case-series)

Does this intervention help? (Treatment Benefits)

Step 4 (Case-series)

What are the COMMON harms? (Treatment Harms)

Step 4 (Case-series)

What are the RARE harms? (Treatment Harms)

Step 4 (Case-series)

Is this (early detection) test worthwhile? (Screening)

Step 4 (Case-series)

## Highlights

• Metastases in cutaneous spinalioma significantly reduce the prognosis.
• Tumor parameters increasing the risk of their incidence are known.
• Elective parotidectomies were performed for high-risk skin head tumors.
• Occult metastases were not found in any of the patients.
• Elective parotidectomy is not beneficial even for high-risk tumors.

## Introduction

Squamous cell skin carcinoma (cSCC) is the second most common human malignant tumor^1^, ^2^ after basal cell carcinoma. In recent years, the incidence of this tumor has been increasing worldwide.^1^, ^3^, ^4^ Presumed causes are the increasing average age of the population, a greater cumulative dose of ultraviolet radiation due to the depletion of the ozone layer, and frequent outdoor leisure activities.

Up to 90% of cSCCs are diagnosed on skin of head, which is most exposed to the cancerogenic agents.^5^ The incidence of cSCC lymphogenic metastases in head region is higher than in other locations but it still does not exceed 10%.^6^, ^7^ The prognosis of patients with cSCC without metastases is excellent after adequate therapy: the probability of a five-year Specific Survival (DSS) and a Disease-Free Interval (DFI) exceeds 95%, otherwise it declines to about 50%.^4^, ^7^, ^8^, ^9^, ^10^, ^11^, ^12^, ^13^ In addition to the high biological aggressiveness of nodal metastases, the reason for such a poor prognosis may also be the fact that after successful treatment of the primary tumor, patients are not always adequately followed, which leads to a delay in metastases detection and therapy.

A negative role can also play the fact that, despite the known risk factors of cSCC lymphogenic spread,^11^, ^14^ prophylactic removal of Lymph Nodes (LNs) is not indicated according to current recommendations due to a generally low incidence of metastases.^2^

The conservative approach is further emphasized by the fact that most of these primary tumors are located in the tributary areas for intra- and paraparotid (hereafter referred to as parotid) LNs, and the potential risks of their dissection by elective parotidectomy may not be accepted by the patient.

The aim of the study is to determine the incidence of occult nodal metastases and evaluate the importance of elective parotidectomy and neck node dissection in patients with cN0 cSCC of a face and scalp.^15^, ^16^, ^17^

## Methods

A cohort of patients treated at ENT clinic of the University Hospital Olomouc over a thirteen-year period (2008–2020) was included in the retrospective analysis. We obtained data from medical records, clinical examinations of patients and evaluation of preoperative Computed Tomography (CT), ultrasound and Magnetic Resonance (MRI) imaging. Resections of the primary skin tumors, the number of the LNs and the presence of metastases, for parotid and neck regions separately, were evaluated by a pathologist. The project was approved by The Ethic Committee of the University Hospital Olomouc and Faculty of Medicine and Dentistry Palacký University Olomouc nº 149/22.

During this period, 117 patients were treated with cSCC of the head. Regional metastases (cN+) were clinically present in 42 patients with neck skin tumors: in 24 of them only in the parotid LNs, in 15 patients in the parotid and the neck LNs, and in 3 patients exclusively in the neck LNs.

In 75 patients, the preoperative clinical and radiological findings on the tributary LNs were Negative (cN0). The stage of tumors, evaluated by the currently valid TNM-8 classification,^18^ was determined by clinical examination, as well as by CT (in some patients also by ultrasonography or MRI) of the parotid gland and neck. In 73 cases, the primary tumors were treated surgically, in 2 patients with radiotherapy.

**Figure 1:**
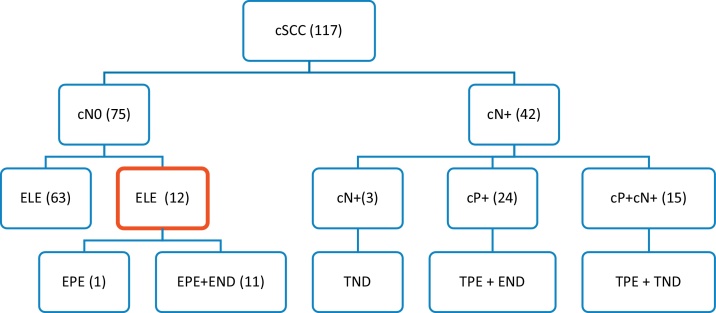
Therapeutic approach to regional LNs in our patients with cSCC of head and neck. cN-, clinical evaluation of regional neck metastases; positive, negative (cN0, cN+); cP+, clinical evaluation of parotid metastases; positive, negative (cP0, cP+); ELE, elective regional LNs dissection; EPE, elective parotidectomy; END, elective neck dissection; TPE, therapeutic parotidectomy; ND, therapeutic neck dissection.

A total of 12 cases were included in the study, of which 4 were local tumor recurrences and 8 were primary tumors. Eleven patients underwent elective surgery on the parotid and cervical LNs. Elective parotidectomy only was performed in one patient (Fig. 1). The elective parotidectomy was indicated in 8 patients as a surgical approach ensuring radical resection of the skin tumor with sufficient margins of the tumor, while simultaneously visualizing the course of the facial nerve. In remaining 4 patients, the indication was based on clinically high risk of the tumor for metastatic spread.

## Results

The cohort of 12 patients consisted of 11 men and 1 woman aged 63–88 (average 70) years (Table 1). Four tumors were located on the skin of the auricle, 3 on the external nose, two on the external ear canal and temporal region each, one on the face. Two patients had one, and the other ten had two or more risk factors for nodal metastasis according to the NCCN^2^ (i.e., tumor stage above T1, deep tumor invasion more than 4 mm, tumor recurrence, G3 histopathological grade) (Table 2). Elective superficial parotidectomy was performed in 10 patients, and total parotidectomy in 2 patients. With the exception of one patient, the elective procedures were also performed on the neck LNs, of which in 9 cases in level II, one patient each in areas II and III, resp. II, III and V. In parotis 3–7 (mean 4, median 3.5) and in neck 3–27 (mean 7, median 7) LNs were identified.

**Table 1:** Overview of patients, characteristics of skin their tumors and elective procedures on parotid and neck LNs.

Patient, sex, age	Risk factors of skin tumor	Grade	Skin tumor location	T stage	Contemporary status (duration in months)	Dissected neck regions (number of LNs)	Type of parotidectomy (number of LNs)
SČ, m, 81y	DI	G 2	Auricle	T2	CR – 58 m	II (4 LN)	PPE (3 LN)
FJ, m, 68y	DI, R	G 3	Nose	T1	CR – 10 m	II (3 LN)	PPE (3 LN)
JK, m, 73y	DI, R	G 2	Ear canal	T3	CR – 64 m	II, III (27 LN)	PPE (4 LN)
ZK, m, 70y	DI	G 2	Auricle	T2	CR – 51 m	II (4 LN)	TPE (7 LN – superficial lobe)
PK, m, 70y	DI	G 2	Temporal	T3	CR – 35 m	II, III, V (22 LN)	PPE (4 LN)
JM, m, 64y	DI	G 3	Ear canal	T1	Recurrence in skin 35 m, hereafter CR 120 m	II (4 LN)	TPE (4 LN – superficial lobe)
PM, m, 69y	DI	G 2	Nose	T1	CR – 50 m	II (4 LN)	PPE (3 LN)
ZO, m, 63y	DI	G 3	Auricle	T1	CR – 48 m	II (4 LN)	PE (3 LN)
IP, m, 71y	DI	G 1	Auricle	T2	CR – 3 m	II (3 LN)	PPE (3 LN)
AR, m, 88y	DI	G 1	Temporal	T3	CR – 5 m	II (5 LN)	PPE (7 LN)
AV, m, 74y	DI, R	G 2	Face	T2	CR – 6 m		PPE (4 LN)
JV, m, 70y	DI, R	G 3	Nose	T3	CR – 121 m	II (4 LN)	PE (3 LN)

DI, deep invasion of the tumor ≥4 mm; R, recurrence of the skin tumor; CR, complete remission; PPE, partial parotidectomy; TPE, total parotidectomy; I, II, III, IV, V, areas of neck LNs.

**Table 2:** Characteristics of skin tumors and overview of risk parameters of lymphogenic spread of primary cSCCs of the head.

Skin tumor location	Auricle	4
	Ear canal	2
	Nose	3
	Temporal	2
	Face	1
T stage	T1	4
	T2	3
	T3	5
Grade	G1	2
	G2	6
	G3	4
Skin tumor recurrence		4
Depth of tumor invasion	≥4 mm	12
Parotidectomy		12
Number of LNs (median; mean)		3–7 (3.5; 4)
Neck dissection		11
Number of LNs (median; mean)		3–27 (4; 7)

The presence of metastasis was not proven in any of the evaluated LNs by histopathological examination.

In both patients after total parotidectomy, transient grade 2–3 facial nerve palsy (according to House Brackmann [HB] classification) developed. We did not experience any other complications such as postoperative salivary fistula, Frey's syndrome or first-bite syndrome.

Only a single patient with cSCC of the external ear canal developed a local recurrence 35 months after surgical resection. After resection of the recurrence, the patient is in complete remission, i.e. without clinical evidence of regional metastases. Regional nodal relapse was not detected in any patient after the elective lymphadenectomy. No one has died in relation to cSCC. The follow-up interval ranged from 3 to 157 (median 48) months.

## Discussion

None of our twelve patients with cN0 cSCC of the head developed an occult metastatic involvement of the resected parotid LNs. This finding is in agreement with the experience of Osborn,^19^ whose study included only a small group of 11 patients after elective parotidectomy. Comparable results were reported by Hoch, who in a prospective study of 13 patients detected occult parotid metastasis in only one patient with locally advanced tumor.^20^

A higher, 16% and 19% incidence of microscopic nodular involvement of the parotid LNs was found in similarly large sets by Kampel et al.^21^ and Wong and Morton.^22^ However, the primary tumors in these cases showed factors associated with a higher risk of lymphogenic spread of the tumor. These authors, like us, do not consider prophylactic parotidectomy to be indicated, as the risk of occult involvement of the LNs in their sets did not reach the consensual limit of 20%, which is the indication for elective lymphadenectomy in SCC of the upper aerodigestive tract.^23^

In contrast, Xiao et al.^24^ and Kadakia et al.^25^ prefer elective parotidectomy for advanced (T3/4) and/or perineurally and intravascularly spreading and poorly differentiated tumors. In their cohorts of patients, subclinical parotid metastases were demonstrated in 20% and 23% of cases. In their opinion, the presence of these risk factors should indicate an elective parotidectomy.

In our group, high-risk tumors behaved comparably to tumors without increased risk parameters. Summing up, so from our point of view, we do not find a reason for elective parotidectomy even in these high risk tumors.

A very high, 54% and 100% risk of parotid occult metastases was demonstrated by Ch’ng et al.^11^ and Kampel et al.,^21^ in patients with clinically evident metastases to the neck LNs, which represent the second echelon of lymphogenic spread of these tumors. In this case, the indication for elective parotidectomy is obvious. This sequence of lymphogenic spread is also confirmed by our cohort, in which none of the metastatic cSCCs of the head had exclusively neck metastases without involvement of the parotid gland.

Similar to the case of elective parotidectomy, the opinion on the simultaneous dissection of neck LNs in cN0 cSCC of the head is also ambiguous. In our group of 12 patients, we performed this procedure in 9 cases, always with a negative histopathological finding. Dür detected occult metastases in parotis in 2 cases, however, subclinical neck metastases were not demonstrated in any case.^26^ Sweeney found occult nodal involvement in only 5% of cases in which the result of elective parotidectomy was negative.^27^ Both authors, like us, are therefore of the opinion that elective neck dissection of neck LNs is not indicated in patients in whom microscopic parotid metastases have been excluded. However, this procedure is justified when parotid metastases are detected, as in these cases subclinical neck metastases occur in 20%‒80%.^24^, ^26^, ^28^

**Figure 2:**
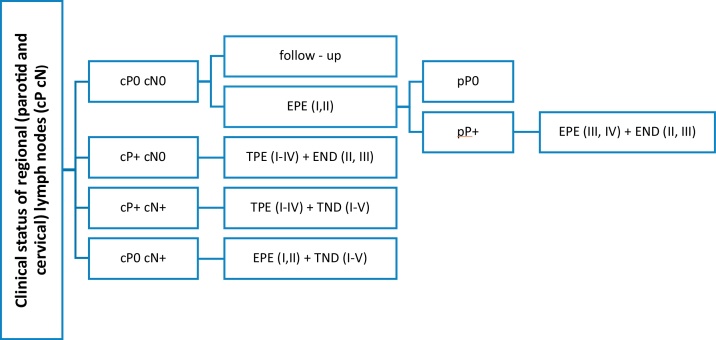
Therapeutic suggestions for regional LNs in patients cSCC of the head and neck. cP+, clinical evaluation of parotid metastases; positive, negative (cP0, cP+); cN-, clinical evaluation of regional neck metastases; positive, negative (cN0, cN+); pP+, pathological evaluation of parotid metastases; positive, negative (pP0, pP+); I–IV, parotid or cervical regions; EPE, elective (partial) parotidectomy; TPE, therapeutic (total) parotidectomy; END, elective (selective) neck dissection; TND, therapeutic (comprehensive) neck dissection.

Based on literature review, it is evident that there is no general consensus on elective parotid node surgery for cSCC of the head. The presence of known risk factors for the lymphogenic spread of these tumors does not represent a clear indication either, as the studies that demonstrated 20%, or even a higher incidence of occult parotid metastases, were retrospective and were burdened by patients selection bias.^24^, ^25^ However, this procedure must be considered in patients with a positive finding in the neck LNs, as the parotid LNs are sentinel ones and leaving them in place would increase a risk of a residual tumor. The extent of eventual elective parotidectomy is based on anatomical studies. Most of the parotid LNs are located in the superficial lobe and periglandularly, while their number is minimal in the deep lobe of the gland.^29^, ^30^, ^31^ This is also consistent with the findings in our two patients who underwent total parotidectomy. No LNs in the deep lobe were microscopically identified. The legitimacy of only the superficial lobe resection is also supported by the clinical experience of other authors^21^, ^26^ in whose small sets, the deep lobe LNs were not positive in any of the cN0 patients. Total parotidectomy is appropriate in the case of clinically confirmed positive parotid LNs, in which the risk of involvement of deep lobe LNs according to Thorn and Kampel is 23%, respectively 20%^21^, ^32^ (Fig. 2).

The dilemma of elective surgery on LNs might be solved by identifying the sentinel LN, which is a routine method in skin malignancies of the trunk and limbs. In the case of head and neck tumors, it is usually not performed due to the significant anatomical variability of the lymphatic vessels, which can complicate a correct identification of a sentinel LN. Another limitation is the small anatomic region where the activity of the radiopharmaceutical applied in the vicinity of the primary tumor frequently overlaps that of the sentinel LN in the parotid. In addition, the extirpation of an isolated selected LN in parotis is of a high risk of facial nerve injury as the nerve passes through the gland and it may be difficult for a surgeon to protect its branches without dissecting the whole course of the nerve.^33^, ^34^, ^35^

Interestingly, numerous studies on histopathological and molecular markers of lymphogenic spread of cSCC (VGFR, PDL, miRNA) are performed. Their prognostic and predictive significance for selection of patients suitable for more radical therapy could provide individually tailored indications for elective dissection of regional LNs.^36^, ^37^, ^38^

## Conclusion

It is clear from a number of published studies that parotid, synchronous, as well as metachronous metastases of the head cSCC represent a significant negative prognostic factor.^4^, ^7^, ^8^, ^9^, ^10^, ^11^, ^12^ However, the importance of the rarely performed elective parotidectomy in these malignancies has not been evaluated in any sufficiently large randomized study yet.

Current international guidelines^2^ do not recommend this procedure even in cases in which the primary skin tumor bears risk factors for nodal metastases. This opinion is also supported by our study: at least one risk factor was present in the primary tumor of all patients with Negative regional LNs (pN0). A lack of specificity and low positive predictive value of these risk factors prevent their use as indicators for elective dissection of regional LNs. In addition, all so far published studies are mostly burdened by a small number of patients.

## Funding

The project was supported by 10.13039/501100003243Ministry of Health of the Czech Republic – conceptual development of research organization (FNOl, 00098892) and internal research IGA LF 2023-08.

## Conflicts of interest

The authors declare no conflicts of interest.
